# Do women with a history of breast cancer recommend risk-based breast cancer screening? An in-depth interview study

**DOI:** 10.3389/fpsyg.2025.1414099

**Published:** 2025-04-22

**Authors:** Zi Lin Lim, Freda Giam, Renee Ying Xuan Wong, Jonathan Jun Kit Liow, Keri McCrickerd, Jingmei Li

**Affiliations:** ^1^Laboratory of Women’s Health and Genetics, Genome Institute of Singapore (GIS), Agency for Science, Technology and Research (A*STAR), Singapore, Singapore; ^2^Human Development, Institute for Human Development and Potential (IHDP), Agency for Science, Technology and Research (A*STAR), Singapore, Singapore; ^3^Department of Paediatrics, Yong Loo Lin School of Medicine, National University of Singapore, Singapore, Singapore; ^4^Department of Surgery, Yong Loo Lin School of Medicine, National University of Singapore, National University Health System, Singapore, Singapore; ^5^National Cancer Singapore, SingHealth, Singapore, Singapore

**Keywords:** risk-based screening, mammography, qualitative, breast cancer, Singapore, genetic test

## Abstract

**Objectives:**

Personalizing screening recommendations could enhance efficiency, support timely detection, and optimize resource use. This study explores women’s perceptions of the facilitators and barriers to current screening guidelines and the implementation of risk-based screening (RBS) for breast cancer in Singapore.

**Methods:**

Individual semi-structured interviews were conducted with 11 women aged 21 and above with a history of breast cancer. Data coding and thematic analysis were guided by the Health Belief Model (HBM).

**Results:**

Five themes were identified and mapped to the Health Belief Model (HBM): (1) Knowledge and beliefs, (2) Access to mammography screening, (3) Social influences, (4) Healthcare delivery, and (5) Needs and preferences for RBS implementation. Key barriers to screening adherence included low perceived susceptibility, cost concerns, and accessibility issues. Factors that could improve adherence included social influences promoting breast health awareness, reminders from trusted healthcare professionals (HCP), and confidence in affording screening and treatment. Participants were generally receptive to RBS and valued personalized recommendations, but concerns were raised about risk prediction accuracy, insurance implications, and potential negative reactions to risk results.

**Conclusion:**

This study identifies challenges and enablers for enhancing breast screening in Singapore, based on the experiences of breast cancer survivors. Participants supported RBS for routine screening. Successful RBS implementation requires improved health literacy, HCP engagement, and accessible healthcare. Women’s acceptance will rely on research to refine prediction accuracy and communication of risk results.

## 1 Introduction

### 1.1 Background

Breast cancer is the most common cancer among women worldwide. In 2020, there were over 2.3 million new cases, and this number is predicted to exceed 3 million by 2040 (Arnold et al., 2022). In Singapore, approximately 465 women die from breast cancer each year (Health Promotion Board Singapore, 2022).

Early detection of breast cancer has been shown to reduce mortality and improve disease prognosis (Duffy et al., 2020). These methods include clinical breast examination, ultrasound, MRI, and blood tests (Wang, 2017; Loke and Lee, 2018; Eisemann et al., 2025; van Winkel et al., 2025). Mammography is known as the most reliable and valid tool for early detection of breast cancer (Ghoncheh et al., 2016; Coleman, 2017) and widely used as part of national breast screening programs around the world (Trieu et al., 2022; Wilkinson et al., 2025).

In Singapore, national screening guidelines recommend that those aged 40 to 49 discuss mammography screening with their healthcare provider (HCP) and consider annual screenings. For women aged 50 and above, biennial mammograms are advised (Ministry of Health Singapore, 2010). Despite efforts to promote mammography accessibility (Health Promotion Board Singapore, 2024), only 37.6% of Singapore women aged 50 to 69 years had attended a mammography screening in the past 2 years (Ministry of Health Singapore, 2022). This mammography uptake is relatively low for a high-income country. In European countries with organized screening programs, participation rates can be as high as 80% (Sterlingova et al., 2023; Eurostat, 2024). Many women perceived screening as unnecessary because they felt healthy (Ministry of Health Singapore, 2022).

Studies have identified several barriers to screening. These include a low perceived risk due to the absence of family history or symptoms, reluctance to pay even subsidized costs, concerns about pain, and a fatalistic outlook (Yin et al., 2007; Teo et al., 2013; Goh et al., 2022). At the same time, breast cancer is increasingly being diagnosed in women under 54. Factors such as early onset menarche, delay in childbirth, and an unhealthy lifestyle may contribute to this trend (Chia et al., 2005; Sim et al., 2006; Singhealth Singapore, 2020). The poor breast screening uptake in Singapore must be addressed to improve women’s quality of life and reduce mortality through early detection (Arnold and Quante, 2018; Katz et al., 2018).

### 1.2 Classification of breast cancer risks for women

Breast cancer risk is associated with many risk factors, including but not limited to old age, family history, genetic susceptibility, breast density, and lifestyle choices (Admoun and Mayrovitz, 2022; Bodewes et al., 2022). The discovery that multiple genetic variants from genome-wide association studies can collectively contribute to breast cancer risk, measured as a polygenic risk score (PRS), has led to the rise of personalized breast cancer risk profiling (Mavaddat et al., 2019; Liu et al., 2022).

Risk-based screening (RBS) may integrate PRS with other non-genetic risk factors to improve risk prediction, considering multiple factors together rather than in isolation (Ho et al., 2022; Yang et al., 2022; Ho et al., 2023). This approach helps classify women into risk levels, tailoring screening recommendations to reduce interval cancers in high-risk women (Evans et al., 2012) and exploring supplementary screening options for women with dense breasts, who may receive false-negative results from mammography (Boyd et al., 2007). RBS has also been found to be potentially more cost-effective than traditional age-based screening or no screening at all (Schousboe et al., 2011; Khan et al., 2021). It is being tested in several countries, including the United States (Esserman et al., 2017), United Kingdom (French et al., 2020), Israel, Belgium, Italy, and France (Delaloge et al., 2022). Assessing breast cancer risk before age 50 aligns with established screening principles, yet a substantial number of women at moderate to high risk remain undetected (Usher-Smith et al., 2023).

### 1.3 Breast cancer risk-based screening (RBS) in Singapore

In Singapore, an ongoing study, ‘BREAst screening Tailored for HEr’ (BREATHE), offers interested women aged 35 to 59 years old a chance to undergo breast cancer RBS. An individual’s genetic and non-genetic risks are assessed using a demographic and lifestyle questionnaire, mammography screening results, and buccal swab. Participants in the study receive a personal breast cancer risk report that has a five-year risk classification with customised screening recommendations (Liu et al., 2022). Existing literature on Singaporean women’s perceptions and receptivity towards the implementation of breast cancer RBS is limited. A qualitative study conducted by Wong et al. on Singaporean women’s views towards single nucleotide polymorphisms (SNP) genetic testing found mixed responses in acceptance of the concept (Wong et al., 2017). Information on accuracy, cost, invasiveness, and side effects were found as factors for consideration.

### 1.4 Evaluating needs for implementing breast cancer risk-based screening

Implementing breast cancer RBS on a national level requires a consideration of the collective impact from individual capabilities, motivations, beliefs, and social infrastructures. The Health Belief Model (HBM) is a conceptual framework that helps explain people’s likelihood of engaging in healthy behaviours to prevent or manage illnesses. HBM has been widely used to gather and explain the potential influences behind women’s commitment to breast cancer screening (Todd and Stuifbergen, 2011; Rainey et al., 2018; Ramezankhani et al., 2023).

HBM consists of six key constructs: perceived susceptibility, perceived severity, perceived benefits, perceived barriers, cues to action, and self-efficacy. These constructs consider women’s engagement in breast cancer screening suggest that women’s engagement in breast cancer screening is shaped by their awareness of their own risk, understanding of the disease’s severity, recognition of the benefits of early detection, potential obstacles and external facilitators, and their confidence in taking action (Skinner et al., 2015). This study builds on previous research examining challenges and opportunities in mammography screening and RBS within the general population (Liow et al., 2022a; Liow et al., 2022b; Kamila et al., 2025). By focusing on women with a history of breast cancer and their experiences with different screening methods (e.g., mammography, ultrasound, genetic risk testing), this study aims to identify key needs that should be addressed to support the successful implementation of breast cancer RBS in Singapore.

## 2 Methods

### 2.1 Participant recruitment

We examine the attitudes towards breast screening among women with a history of breast cancer. This work expands on insights from previous studies involving other populations (Liow et al., 2022a; Liow et al., 2022b; Kamila et al., 2025).

**Figure 1:**
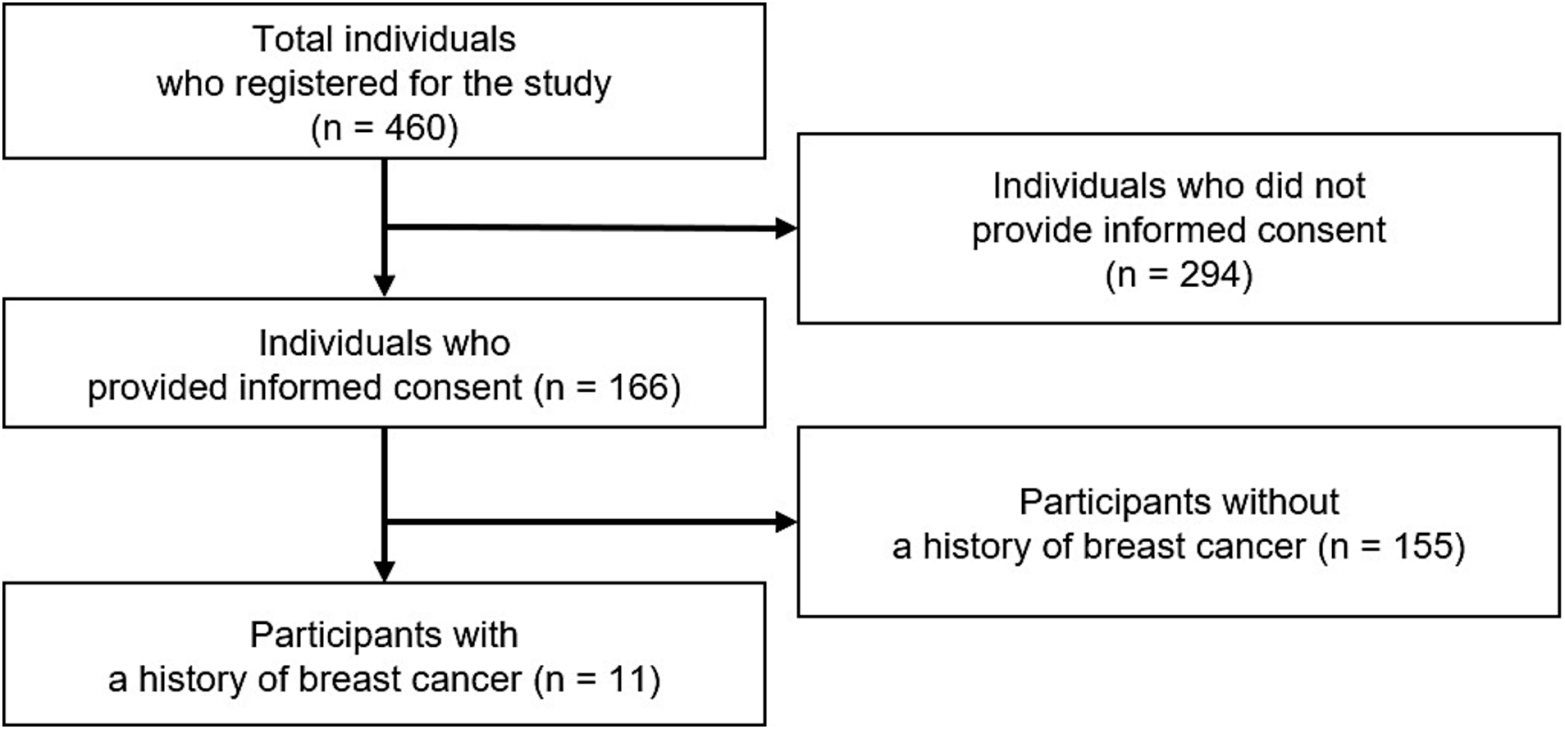
Flow chart of the study population.

English-speaking participants aged 21 and above with internet access were recruited through non-profit organizations via social media and email. None had pre-existing relationships with the researchers. In total, the study received registrations from 460 individuals, of whom 166 participants provided verbal consent via one-to-one online sessions. Among them, 11 women with a history of breast cancer were purposefully selected for in-depth interviews (Figure 1). Data collection ceased once saturation was reached, defined as the point at which no new discussion points emerged (Grady, 1998).

### 2.2 In-depth interviews

A semi-structured interview guide was developed based on existing literature and insights from experts in breast cancer, epidemiology, and psychology. It covered topics such as awareness of breast cancer and screening, motivators and barriers to screening, and perspectives on risk-based screening. A detailed version of the guide can be found in Supplementary Table 1.

Since RBS is still a relatively new concept to the public, participants received a brief introduction during the interview. A risk report prototype was also shown to the participants to gather feedback (Additional file 1).

Each interview was conducted by one of two members of the research team: a 24-year-old Chinese female with expertise in Public Health and Life Sciences (BSc) or a 26-year-old Chinese male, specialising in Psychology and Communication (BA). Their professional backgrounds were relevant to the research topic, helping to facilitate a deeper understanding of issues. During the analysis phase, efforts were made to check for any biases by consulting with other research members. The one-on-one interviews, conducted in English online, took place between September 2022 and December 2022, with each session lasting approximately 60 min. At the end of the session, each participant received a digital voucher worth 60 Singapore Dollars (SGD).

### 2.3 Data analysis

Interviews were recorded in audio-visual format and transcribed verbatim. Participants were de-identified, and each was assigned a unique alphanumerical code (e.g., F1) to ensure anonymity and all confidential information was redacted. The data were analysed through a five-step process:

*Step 1: Familiarisation of data.* Researchers (FG, ZLL, and RYXW) reviewed transcripts thoroughly and noted down their reflections.
*Step 2: Development of codebook.* HBM was used deductively for data categorisation. Data familiarisation of transcripts and the HBM constructs have guided the development of codes and sub-codes under each HBM construct, accompanied by definitions (Supplementary Table 2). The constructs, codes, and sub-codes were further refined through continued discussions among study team members (FG, ZLL, RYXW, KMC, JL). This codebook served as a backbone for coding all transcripts in the QSR Nvivo software package (Version 20.7.2, QSR International).
*Step 3: Piloting codebook.* Three independent coders (FG, ZLL, RYXW) coded the first four transcripts by categorising participants’ responses to the relevant HBM constructs and sub-codes. The coders met to discuss their codes and continued to revise the meaning of all codes and refine the codebook.
*Step 4: Final coding process.* The finalised codebook was used to code the remaining seven transcripts by the same independent coders, and all codes were discussed and resolved.
*Step 5: Thematic analysis.* HBM was used as a fundamental conceptual framework to guide coders in segmenting the data. As the constructs of the HBM framework can be rather isolated in themselves, we have opted to perform further thematic analysis to better explain certain behaviours. This will help identify common themes and actionable insights that encompass various aspects of the HBM. The independent coders did a final review of the codebook and transcripts and individually came up with overarching themes and the relations between them based on the data. Final themes, subthemes, and definitions were decided through iterative discussions between the study team members. The HBM constructs from the codes were eventually mapped against the emergent themes.

This study followed the Consolidated Criteria for Reporting Qualitative Research (COREQ) guidelines with a checklist (Additional file 2) (Tong et al., 2007).

### 2.4 Ethics board and study approval

This in-depth interview study received approval from the A*STAR Institutional Review Board (2021-077), and all participants provided recorded verbal consent before their involvement.

## 3 Results

### 3.1 Population characteristics

Eleven women with a history of breast cancer were interviewed. Overall, the mean age of participants was 43.9 years, and the majority were of Chinese ethnicity (63.6%) (Table 1).

**Table 1:** Characteristics of study population (*n* = 11).

Characteristic	Value
Median age at interview, years (range)	45 (36–52)
Ethnicity, *n* (%)	
Chinese	7 (63.6)
Malay	0 (0.0)
Indian	1 (9.1)
Other	3 (27.3)
Marital status, *n* (%)	
Married/have a partner	6 (54.5)
Single	5 (45.5)
Mother’s history of any cancer, *n* (%)	
Yes	2 (18.2)
No	6 (54.5)
Unknown	3 (27.3)
Family history of breast cancer, *n* (%)	
Yes	0 (0.0)
No	11 (100.0)
Working status, *n* (%)	
Working	9 (81.8)
Non-working	2 (18.2)

### 3.2 Facilitators and barriers to breast cancer RBS

**Figure 2:**
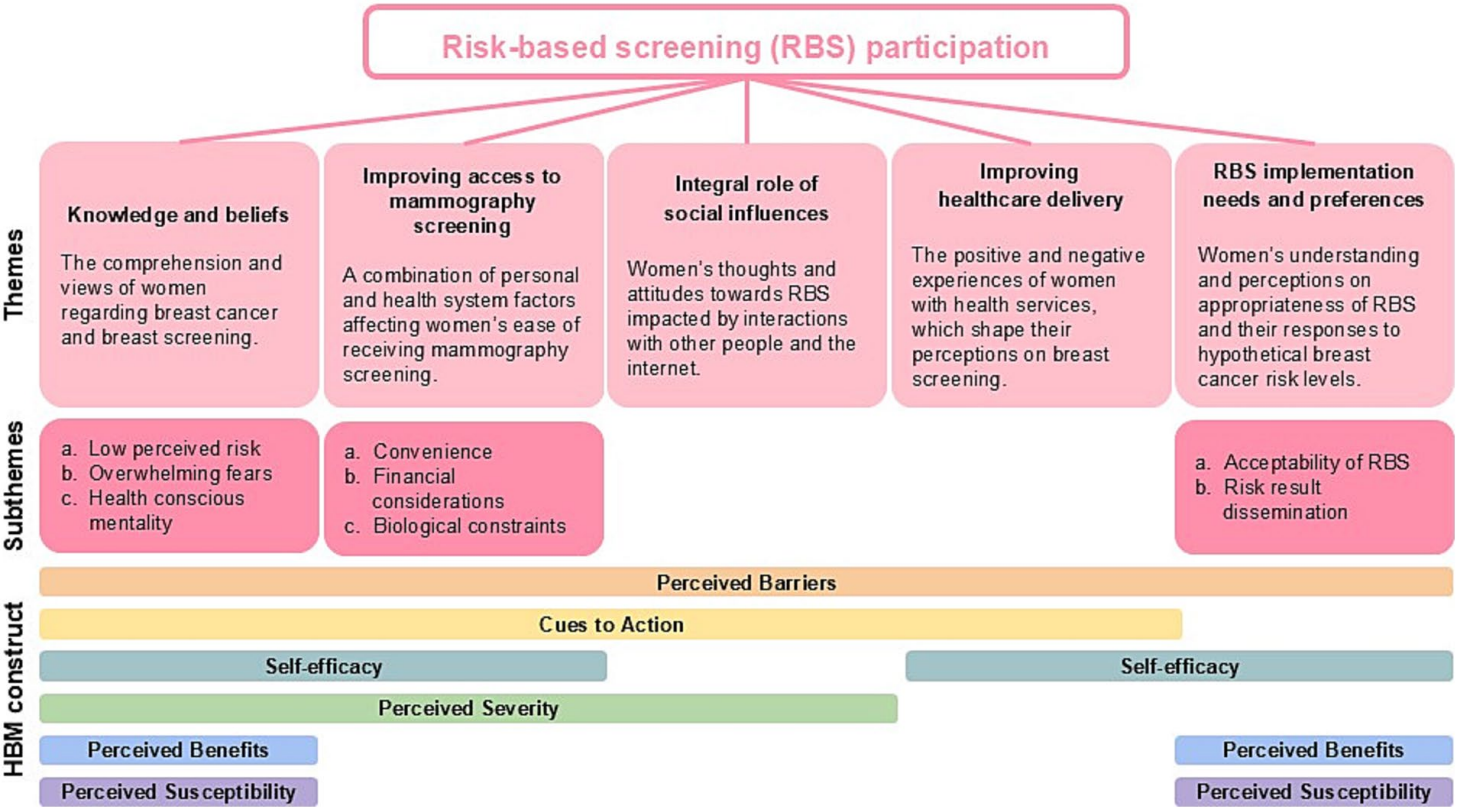
Key themes of women with lived breast cancer experiences and their views on risk-based breast cancer screening.

Results from the 11 interviews revealed five themes for consideration when designing and implementing novel breast cancer risk-based screening for women. They are (1) Knowledge and beliefs, (2) improving access to mammography screening, (3) the Integral role of social influences, (4) improving healthcare delivery, and (5) RBS implementation needs and preferences. These five themes were mapped onto the HBM to help determine types of behavioural factors for modification to increase breast screening health behaviour as shown in Figure 2.

#### 3.2.1 Knowledge and beliefs

This theme focuses on how women’s understanding and perceptions about breast screening influence their decisions to participate in regular screening. It is characterised by three sub-themes: low perceived risk, overwhelming fears, and health-conscious mentality. The theme “Knowledge and beliefs” was mapped onto six domains of the HBM: ‘Perceived susceptibility’, ‘Perceived barriers’, ‘Cues to action’, ‘Perceived benefits’, ‘Self-efficacy’, and ‘Perceived severity’.

##### 3.2.1.1 Low perceived risk

Although participants were found to be well-informed about risk factors contributing to breast cancer (e.g., lifestyle, genetics) and mammography screening, the majority belonged to a younger age group (below 40). Thus, they reflected having low self-perceived risk before their diagnosis. Additionally, other causes of low self-perceived risk include breastfeeding history, lack of breast cancer family history, and self-perceived healthy lifestyle. Their low perceived risk contributed to a lack of proactiveness in screening pre-diagnosis. However, post-diagnosis, participants acknowledged breast cancer’s indiscriminate nature and emphasised the necessity of initiating early conversations about breast health and screening to raise awareness.

*F11: (Did not go for screening often) because I did not really think that I would get cancer … I was, you know, below 40, and we do not have family history of cancer, and then I was eating healthy, and I was active. So I thought I was doing everything I could. I mean cancer was never on my mind…*

*F7: In my case, I had two kids. I do not smoke. I do not drink. I breastfed both kids [for] 19 months. I have breast cancer. I had no symptoms at all…. So perhaps education should start earlier, to tell people that it happens. You cannot think that it will not happen to you.*

##### 3.2.1.2 Overwhelming fears

Another barrier to mammography screening participation was the irrational fears women can have from the discomfort of the mammography procedure and getting diagnosed with breast cancer.

*F10: There were two misconceptions that I had before my diagnosis. One is mammogram is really painful, like no point going through it.*

Pain can vary depending on individuals. Some women found mammography tolerable while others have also explained that while the procedure can be uncomfortable, they recognised the benefits outweigh the drawbacks of screening.

*F10: … different people will have different level of threshold. I thought it was discomfort because they literally squeeze your breast to go into that machine. But honestly, there is only 1 or 2 min of that. If you were to ask me how painful, 1 or 2 min, if anyone had gone through childbirth or anything [the pain from mammography screening should be tolerable]. So to me, it’s actually okay.*

*F8: Can be a bit unpleasant, but I guess, for the sake of detecting any abnormalities I think for that few seconds I just have to put up with it.*

Besides mammography screening being uncomfortable, another common fear is receiving bad news of breast cancer diagnosis. Some women succumb to avoidant behaviour as they cannot put up with the potential aftermath of a diagnosis, such as major lifestyle changes, financial impact of treatment, job insecurity, and identity loss from breast mastectomy.

*F3: I have people who think that if I do not go for check-up, if I do not go for any monitoring, at least I do not know if there’s anything wrong with me, I can still continue in my lifestyle.*

*F7: I also had a few friends whose mothers-in-law would rather die (than go for screening/ treatment) because they outright just told the daughter-in-law 没有胸部不是女人 (translated: no breasts, not a woman).*

##### 3.2.1.3 Health-conscious mentality

Conversely, some health-conscious participants were highly motivated to engage in regular breast screening once eligible stemming from the fear of late detection and determination to live for their loved ones. Their motivation to attend mammography was also partly due to their knowledge and awareness that breast cancer is not a death sentence and early detection can lead to favourable prognosis.

*F7: They (her kids) are still very, very young, and I want to make sure that, you know, we can be around for a long time to see them grow up. And so that’s probably the reason why my husband and I are very, very diligent in getting our annual checkup just to make sure that we have more years with them.*

*F5: I think it [screening] really depends on the person’s character also… I think education is very important and of course, I think we need to highlight [to the public] that you know breast cancer is very highly treatable, it is not something that you know… when you have it… uh you would immediately die.*

#### 3.2.2 Improving access to mammography screening

The factors affecting accessibility to mammography screening can be characterised by three sub-themes: convenience, financial considerations, and biological constraints. The theme “Improving access to mammography screening” was mapped onto four domains of the HBM: ‘Perceived barriers’, ‘Cues to action’, ‘Self-efficacy’, and ‘Perceived severity’.

##### 3.2.2.1 Convenience

Convenience refers to the amount of effort needed to fit mammography screening sessions into women’s lives. The difficulties women face include long travel times to clinics with mammography services, long waiting times before and during appointments, and difficulty remembering appointments due to long intervals. Participants felt that walk-in services and text reminders nearing appointment dates are initiatives that can help improve breast screening attendance.

*F6: [Mammography screening is] very inconvenient. Then you have to do advance booking. So if you can have like a breast screening drop-in center it’s also good… then you do not have to wait like weeks and months to get your appointment.*

*F8: And it’s like quite a long waiting period. Even when you want to go for it, you need to book, then you have to wait quite a long time. So by the time it [the appointment] rolls around it’s like you probably already forgot about it unless you get a SMS reminder, timely reminder. Otherwise it’s like so far in the future. Then you probably miss it already.*

Some participants acknowledged existing services like Mammobus, a mobile breast cancer screening service which aims to bring screening convenience to neighbourhoods. However, for it to be effective, more efforts are needed to publicise important information about the Mammobus’ schedule to build awareness and engage eligible women.

*F5: I know that when you are doing the mobile van, it’s about $10. That’s what my girlfriend told me. But sometimes you are not aware. When is a mobile van coming to your estate? Or where is that?*

Additionally, as the majority of women are working full-time, participants believe that the workplace plays an important role in promoting screening. Participants have suggested companies send out screening campaign details, host mobile breast screening vans in or nearby workplaces, and provide breast screening leave, which gives women extra time to attend screening without sacrificing annual or medical leave.

*F5: So probably just giving them time off, they do not have to take MC (medical certificate)… Or the HR (human resource) can be reminding them “Government has an initiative for colon cancer, breast cancer, cervical cancer, it’s only 5 dollars this month.” I think then it will be easier, because you know everyone spends 8 h a day at workplace. So I’m sure HR would be able to reach out to them better than the government.*

##### 3.2.2.2 Financial considerations

This sub-theme highlights monetary costs, availability of coverage from insurance, and use of financial incentives as factors impacting women’s screening participation.

Participants highlighted how the perceived monetary cost of screening itself can deter women from attending screening.

*F10: Sometimes it may be also cost… because they do not know how much it would cost and I probably think $50 is nothing, but it may mean a lot to some of them.*

*F3: One thing is, it can be quite costly and if you are going through the subsidised route - the public sector route, it can be quite time-consuming. … That’s what I see around me.*

Similar to concerns brought up in the preceding subtheme, some participants felt that more work could be put into educating people on avenues to look for to reduce screening costs.

*F1: You know, finances could be a barrier. But I think for most Singaporeans, it’s heavily subsidised, so educate women about this, that it will cost them nothing.*

Additionally, some participants mentioned that companies offering partial or full subsidies for mammography screening can serve as an incentive to motivate breast screening attendance.

*F10: I just did one recently… So it is partly because my company gave us $350 yearly to go for such a screening, so that actually helps in a way to offset the cost of health screening, …. [it] actually encouraged me to go for the checks.*

While some have considered the initial cost of screening affordable, they may still resist screening due to fear of the financial impact of treatment if diagnosed. Participants with lived breast cancer experience shared how important insurance coverage is in providing women assurance before attending breast screening.

*F10: But I thought that sometimes, because people are also scared of the costs involved after they are diagnosed… So I felt that probably, you know, getting themselves well insured is also very important.*

As the financial impact from treatment upon diagnosis can be huge, treatment and extra screening subsidies upon diagnosis were suggested as potentially useful to encourage breast screening behaviour when the affordability of health expenses upon diagnosis seems less daunting.

*F6: Okay, for example, like, if let us say you go for the scan, and then touch wood, you… kena (get) cancer. Then maybe the treatment, part of the treatment plans, certain portion you know you all can give, give uh, a certain amount of subsidy, or whatever. Or, subsequently, you know the next 3 years or the next 5 years, [let those diagnosed with breast cancer] know that their breast cancer scan will be waived, for example.*

##### 3.2.2.3 Biological constraints

Women’s access to mammograms can be affected by biological constraints such as breast density and menstruation.

Some participants with dense breasts have raised concerns about mammogram’s accuracy in differentiating lumps from dense breast tissues even though mammograms remain as the gold standard for national breast cancer screening in Singapore.

*F2: I was very wary of mammograms… a doctor said something to me like “You have very dense breast tissue. So mammograms are going to be useless, because, like everything looks the same, they cannot differentiate dense breast tissue versus something that’s actually potentially dangerous.” So… that kind of always stuck with me like mammograms are never going to work on me anyway.*

In other situations, women missed their opportunity to screen when their breast screening appointment coincided with their periods, which meant that they would have to reschedule and go through the entire waiting process again.

*F10: … just so happen… there were twice that when I went for such screening, I was menstruating. Then they will tell you that do not do it [mammography screening] because it will not be accurate, or you are not able to do it. So it’s always happened and then I never had a chance to go back and do it again.*

#### 3.2.3 Integral role of social influences

Participants have shared how the presence or absence of breast health conversations among their social circles and information on the internet could impact their decisions to attend breast screening. The theme “Integral role of social influences” was mapped onto three domains of the HBM, ‘Perceived barriers’, ‘Cues to action’ and ‘Perceived severity’.

Participants expressed confidence in becoming health literate as additional information on breast cancer symptoms and mammography screening procedures can easily be accessed online nowadays.

*F4: So I will not say I understand the [mammography] process but at the same time I would say for me right, I can google and find information online so I will not say I do not know.*

Despite this, some participants initially hesitated to undergo screening due to low self-perceived risk. However, exposure to the realities of breast cancer from family, friends, and colleagues influenced their screening motivation. First-hand accounts and experiences of breast screening and/or cancer among acquaintances spurred women to be more proactive in attending breast screenings.

*F4: So from Google, the first few results were that it was breast cancer. I thought… “cannot be.” Then, after that I actually have a colleague who had breast cancer. She’s in her 50s, I think… So I actually asked her. At the time she just went through cancer treatment, so she’s quite familiar. And she told me “Oh, I think you better go and check…”*

*F7: My mother has a pretty big cup [size], and then she has a regular cyst developing in her breast, every now and then. And they are fairly big. So in my family we have talked about mammography quite often, so I already have an idea about it, because my mom goes for it.*

A participant shared that openly discussing her breast cancer journey on social media was effective in advocating for breast health and reducing negative perceptions towards breast screening.

*F10: I shared on my social media, my cancer journey, so that actually kind of inspired or motivated most of my girlfriends to actually go for the screening. Because it [my diagnosis] was so sudden that they also got shocked. And because all my friends who follow me on social media are around my age, and my students also follow me, they also urged their moms to go for screening… So they said that sometimes, they just need someone to really remind them or motivate them to do something… I actually shared my journey, like how mammography works, and it’s actually not that bad. … So I think that actually helps people debunk this myth that it’s painful, and to go for it [mammography].*

Although social circles can positively influence women’s participation in breast screening, some women identified that family upbringing also contributes to one’s mindset and decision on attending regular breast screening.

*F6: … let us say, your parents are the highly educated ones, they would have already inculcated the educated people kind of thinking whereby you will be very… you will be taught to be self responsible for your own health. But if you come from a family background where your parents are… not the educated type, their thinking would be: “Nothing wrong why you go to hospital? Nothing wrong why you go take blood? Nothing wrong why you go screening?” You know, so that is the kind of upbringing you have right, then, over the years you will not have that kind of thinking of wanting to go and do health screening. So I think it’s the environment and also the people you mix with.*

#### 3.2.4 Improving healthcare delivery

Women interviewed shared a mixture of enabling and disabling encounters in healthcare service that could influence their attitudes and involvement towards breast screening. The theme “Improving healthcare delivery” was mapped onto three domains of the HBM, ‘Perceived barriers’, ‘Cues to action’, and ‘Self-efficacy’.

Some participants have positive sentiments towards Singapore’s healthcare system, reflecting trust and pride in the quality-of-care Singapore can provide to people.

*F5: I have quite… large amount of trust and … confident in Singapore[‘s] healthcare system lah. Ya. So… I would say… The results are generally should be dependable, ya.*

*F7: Uh, because it’s in Singapore I think I trust it a lot more. But yeah, I would say that in Singapore I do trust our standard of health care quite a lot, and I really trust the doctors that I go to…*

In addition, participants interviewed have mostly regarded doctors as someone they trust and depend on to explain their health reports and recommend further actions. Other health professionals, such as nurses, were considered the next reliable professionals that people look for to understand their health.

*F11: It’s always helpful, you know, after I get the report I talk to my doctor, and you know I ask them whatever questions I have in mind. So it’s always the report, plus the explanation of the doctor and him or her answering whatever questions I have…*

*F4: Maybe sometimes doctors are busy….in the process there could be someone, be [it] a nurse, to just let the patient ask questions or maybe sit down and really in-depth go through the medical report.*

Women interviewed have made comparisons between healthcare institutions, with the impression that public health institutes may deter some women from attending breast screening due to the nature of its services. Participants perceived healthcare workers in public institutions as task-oriented while those in private institutions were perceived as more people-oriented. Furthermore, some women prefer to have all their health records cared for by the same doctor as they think that seeing different doctors may compromise the quality of healthcare.

*F4: … the people at [private institute] … is so warm… when you come in they welcome you warmly, they offer you hot beverage. So they really explain “So this is what I am going to do…” and they were very gentle with it. Whereas at [public institute] right, it is more clinical, because I guess… there is more people going la… they have a checklist, they ask you questions then … send you there then ok done.*

*F7: … for some of us, we want a consistent record with one doctor. The problem,… in my opinion, about going to … polyclinic, all this is, the doctors will keep changing, because … doctors will rotate shifts… So you do not always have the same guy seeing you, and to be fair to the doctor, he cannot tell you more than what he reads. So it depends on how good the previous doctor was in documenting your situation.*

Besides differences between health institutions, receiving different screening advice from different doctors was also a source of confusion and frustration for some participants. Poor clinical judgement from doctors can lead to unnecessary overscreening or undetected disease. Such problems can bring about scepticism and lack of trust in healthcare, which will deter women from attending breast screening.

*F6: … I was misdiagnosed actually… I ask the doctor to give me mammogram, or you know, ultrascan. But then he say no need, so I think the doctors also need to be educated much as they are given the guidelines, you know, women with lumps, and whatever actually with or without lumps 40 years old and above should go and do mammogram at least or ultrascan, but he flatly rejected me… so the screening process… is the responsibility … not just on the patient itself. It’s also doctors need to be educated, and doctors need to follow the SOP (standard of procedure)…. And especially if this woman,….come and see you… already got a lump,… you should also ask the patient to go and do mammogram or ultrascan.*

Apart from clinical judgement, doctors’ words are highly impactful to patients. Thus, they have to sensibly communicate accurate health information and ensure patients have adequate understanding. Failure to explain clearly could potentially bring about unnecessary worry or undesirable attitudes towards breast screening.

*F7: I think it’s how you educate people to say that because it (a lump) exist, there’s a possibility. Then you have to regularly come back and check. Do not slack off. So it’s how the message is relayed to the individual, and I think it’s not that easy. The doctors also have to see the personality of the patient that they are talking to… if you over-scare them you could turn them off because the girlfriend that wanted to delay her surgery the first doctor that she went to… apparently unloaded so much possibilities on her. It freaked her out… So I think a lot rests on how the doctor communicates with you…*

Although mammography is known as the gold standard for detecting breast cancer, some participants believe that mammography alone is insufficient because of their experiences with undetected tumours from mammography that was picked up by ultrasound.

*F6: … mammogram itself alone is not enough… because if the lump is located at the higher area … on the chest right, then mammogram will not be able to pick it up… unless you do ultrasound, then only you can see. So screening right, should not be mammogram only. It should be mammogram plus ultrascan.*

#### 3.2.5 RBS implementation needs and preferences

The theme “Risk-based screening implementation needs and preferences” was mapped onto four domains of the HBM, ‘Perceived barriers’, ‘Self-efficacy’, ‘Perceived benefits’, and ‘Perceived susceptibility’. Participants’ perspectives on viability of breast cancer RBS national implementation were explored and categorised under two sub-themes: (a) Acceptability of risk-based screening and (b) Risk results dissemination.

##### 3.2.5.1 Acceptability of risk-based screening

When discussing the feasibility of nationwide implementation, participants reflected mixed reactions. Supportive participants expressed that knowing their breast cancer risk would make them more vigilant and facilitate informed decisions such as changing their diet or lifestyle and going for more regular screening.

*F5: If I myself know I’m at risk of certain cancers then I would definitely improve my lifestyle … If I was smoking then I will stop smoking… If I’m obese then okay I will start exercising… I’ve seen people who smoke their whole life and then they get cancer and then they can just stop smoking. But whereas for the past few years you have been trying to stop [them from] smoking but they do not. So probably this can be one of the reasons to help people have better habits when it comes to lifestyle.*

*F8: I would want to know if, like I was at higher risk than not [know]. At least I know to be vigilant about whatever that I’m higher risk for, right?*

Other participants cautioned against potential pitfalls in nationwide RBS implementation, for example, the constant fear of developing breast cancer after knowing one is at above-average risk. It was expected that not everyone would positively react to their risk results which questions the suitability of RBS at a population level.

*F5: I think it really depends on the person’s mentality but personally… (pause) I do not think I would want to know?… But of course I will definitely encourage and I would also do it myself if there’s a very strong family history or [if] the doctors think that we are at higher risk. Because I feel that if you were to introduce this to the general public,on a wide-scale basis, I feel that it would cause unnecessary alarm.*

*F10: This (inclusion of genetic risk in report), I thought it [should be] optional, because you are already going through so much….Some people will be very affected by the fact that “I have it (positive genetic risk), then how?”*

While RBS’s genetic test (polygenic risk score) is of a different nature than what some of these participants have undergone (Oncotype DX, BRCA gene test), participants expressed concerns on the potential additional expenses incurred with genetic tests in screening programs.

*F8: But the one (genetic test) that I underwent was super expensive. It’s like SGD1000, and there’s no subsidies for it. So that cost alone, if let us say I had not been diagnosed, I would not have gone for it just to find out my risk. So the cost must be much lower.*

*F10: I feel that the cost of it is also something that would deter people to go for this genetic testing unless necessary.*

Besides that, some participants were also worried about insurance implications. They questioned protocols involved in the data retrieved and emphasised that data security or other protective measures must be established such that women would not be liable (in the form of higher premiums) if they received an “above-average risk” result.

*F5: I’m not sure how it (insurance) will change especially when it comes to such a test. Will the insurance company then not cover ladies with above-average risk if they were to get breast cancer? And because they (insurance companies) are private [companies] right so it is really up to them whether they want to change their rules. I mean of course MOH (Ministry of Health) can step in but this is another thing that may deter ladies from having such screening.*

*F2: I’m just not in favor of giving insurance companies that knowledge and potentially discriminating, based on things like that, you know… Maybe I’m just skeptical of insurance companies. I think they would use it [the information from RBS] in the wrong way.*

Most participants expressed a greater inclination to trust and participate in RBS when they perceive extensive research backing it, observe high accuracy in its implementation, and receive recommendations from reliable HCPs.

*F9: So I mean that that information must be, you know, trustworthy… How accurate is it? So, I mean, if let us say it is something that is, you know, the accuracy is maybe like 50–50, then I will have my reservation about doing such a check.*

*F5: I think it’s [having different recommendations] fine I mean as long as this is recommended by the doctor and there’s enough information, enough research that has been put into this test I think it’s fine.*

##### 3.2.5.2 Risk result dissemination

This sub-theme covers participants’ feedback on the prototype risk report and suggestions on how risk reports should be effectively utilised in RBS implementation to support breast cancer screening adherence.

Most participants considered the inclusion of personalised risk classification with tailored recommendations and actionable steps in the report useful.

*F10: I thought, this is good. This [the result] is actually a good indicator to let them know [about their risk]…. They give you a whole list of your results, and recommendations.*

*F9: The other points like, you know, how to live healthily, stop alcohol and things like that. So, those are good information.*

But some have found the prototype risk report too wordy and suggested streamlining information or relying more visual aids to improve the report’s interface.

*F5: I think it’s a bit wordy, honestly, I do not think people will read it. Like that much. I think … the number of words can be reduced, anyway they will see a doctor right? So I think it should be kept very simple. But I do like the graphics when it comes to how you can improve…*

Although the prototype risk report has tailored recommendations, some participants expressed that the current prototype lacks additional resources and information to convince and prompt regular breast screening. Suggestions include the addition of QR codes or weblinks to access additional information on how women’s risk was derived, especially for those with above-average risk, appointment system to facilitate follow-ups, and support groups for those who hope to connect with people having similar experiences.

*F11: Uh, so the ones that I see here are more action points right like. Get a one-stop breast care. Go for routine breast self exam.… when you get your let us say breast cancer risk…. I want to know why, why am I above-average? … Let us say I was scared to see that I’m above-average, but when I see that okay, these are the reasons why I got this rating, then maybe that would encourage me to do the action points that are seen in the report.*

*F7: Similarly, your hospital there (referring to recommendations for follow-up), you might want to give them a contact number, might want to give them an appointment booking QR code. Something that will get them through to the number. Nothing is more irritating than a general hotline that nobody picks up, yeah.*

*F1: I know for example the breast cancer foundation has different support groups, different befrienders… You know I think sometimes people do not want to talk to doctors and want to talk to someone who has been in a similar position… In these additional recommendations we should include one more point that says, if you require support, you can [receive support]. Like if someone is above-average risk and wants to talk to someone who has been diagnosed, there’s that option.*

To promote regular breast screening using risk reports, some suggested offering the report in multiple languages to ensure women with limited English proficiency can interpret it accurately and respond appropriately to the recommendations.

*F7: So it’s the mass public that we are looking after, and the mass public may not have that same level of exposure and education, especially for those that are not so strong in English. So a brochure that’s just in English might be daunting. If you have it in the other languages, it’ll be a lot more helpful.*

To prevent undesirable responses such as negligence from below-average risk results or fear from above-average risk results, participants stressed the importance of emphasising that risk results are not absolute and can change with time. This can be achieved by managing expectations before RBS, including disclaimers in the report, and involving HCPs in relaying the risk report results. Participants believe that HCPs have a role to play in managing risk communication and translating medical information for laymen to understand their health more effectively.

*F5: So I think the person before going through this screening report… I think the doctor or whatever, may not be a doctor, but must be able to, probably do some counselling, just to let them know what are they going to expect from this.*

*F10: This [a disclaimer on the predictive function of risk results] one-liner is important to not scare them: It does not mean that they have above-average, that they will get [diagnosed], … below-average does not mean they will not get [diagnosed]. So [the risk] may change over time.*

*F8: Actually the mammogram report (referring to the RBS prototype report) is more for like, the specialist to explain to us. Because most of the reports is using jargon, so a layperson will not understand fully what it’s about. So we still need the doctor, the specialists, to explain to us thoroughly… however detailed or not detailed the report is.*

A few believe that a safer approach is to not have a “below-average risk” result category at all to prevent health complacency.

*F6: So actually, by putting below-average right is actually deterring people from doing (going for regular screening). Because people will very conveniently think that oh, that means my chances of getting it is very low. Then no need [to screen]. Wait until the lump come out then I go [for screening]. Definitely, a lot of them will have this kind of thinking… Shouldn’t, should not put below-average.*

## 4 Discussion

This study identified key obstacles and enablers influencing women’s access to screening in Singapore, providing insights for RBS implementation. Interview findings suggest that screening decisions are shaped by women’s knowledge, beliefs, and their physical and social environments. For RBS specifically, concerns were raised about prediction accuracy, financial implications, and the communication of risk results.

### 4.1 Overcoming perceived barriers

The construct “Perceived Barriers” in HBM mapped onto all identified themes, and multiple existing barriers to screening. Consistent with existing literature, poor accessibility was a common concern, with financial considerations being the primary factor influencing screening attendance (Azami-Aghdash et al., 2015; Sarma, 2015; Malhotra et al., 2016; Momenimovahed et al., 2020). Additionally, financial considerations extended to a fear of undesirable results, as individuals worried about the potential financial burden of a diagnosis (Momenimovahed et al., 2020; Biddell et al., 2021; Miles et al., 2022; Rajendram et al., 2022).

#### 4.1.1 The impact of insurance on screening motivation

Interestingly, participants noted that having insurance provided them assurance for screening, knowing they would be covered if abnormalities were found. A study by Biddell et al. found that among women underscreened for cervical cancer, those with perceived financial barriers often overestimated the potential screening and treatment costs and were unaware of assistance programs (Biddell et al., 2021). Other studies have found that in combination with reduced screening costs, educating communities about currently available screening programs and screening importance are some measures that can be taken to help increase screening rates among low-income groups (Wee et al., 2012; Lin et al., 2021; Rajaguru et al., 2022). Furthermore, when diagnosed early, breast cancer patients are often offered a wider range of treatments, which includes more affordable ones (Blumen et al., 2016; Sun et al., 2018; McGarvey et al., 2022). Highlighting the benefits of early detection and the subsidies available pre- and post-diagnosis can help alleviate some concerns and motivate those who are disadvantaged by financial constraints.

#### 4.1.2 Personalising breast screening for individuals

Asian women have denser breasts than their Western counterparts (Maskarinec et al., 2001a; Maskarinec et al., 2001b), which can contribute to difficulties in obtaining clear images and may lead to false negatives or inconclusive results (Maskarinec et al., 2001a; Maskarinec et al., 2001b; Kerlikowske et al., 2011; Bae et al., 2014; Lee et al., 2017; Mendat et al., 2017). Recognizing these challenges, participants highlighted the need for supplemental screening methods. The use of multiple screening modalities has also been investigated in other studies (Buchberger et al., 2018; Bakker et al., 2019; Vourtsis and Berg, 2019). With the shift towards personalised screening, there is a need to tailor screening approaches and optimise diagnostic tools for improved accuracy across demographic groups.

### 4.2 Improving breast cancer screening prioritization through increased perceived susceptibility

Another barrier identified was women’s low priority for attending screening, a recurrent theme in previous research (Azar et al., 2022; Surendran et al., 2023). Findings have shown that the lack of screening prioritisation is influenced by low self-perceived risk (Oh et al., 2017), a trend similar to that observed among participants in this study. Some women may mistakenly believe that their young age or absence of a family history exempts them from potential risks, leading to a less proactive screening approach (Katapodi et al., 2004; Haber et al., 2012; Dey et al., 2015). Therefore, to better assess one’s risk, it is important to inform women that breast cancer is multifactorial and not solely determined by family history and/or age.

### 4.3 Leveraging cues to action

The construct “Cues to Action” in HBM commonly mapped with the themes found in this study also suggests that multiple points of interventions have helped or can be capitalised on to improve screening attendance.

#### 4.3.1 Empowering conversations: the impact of provider recommendations on screening adherence

Participants in this study indicated high regard for physicians’ recommendations. Combined with high trust in Singapore’s healthcare system, they will often be convinced to attend screening when proactively reminded. Such attitudes have also been echoed in previous studies on the importance of the doctor-patient relationship (Seetoh et al., 2014; Lim et al., 2015; Wee et al., 2016). A systematic review by Peterson et al. showed a positive association between provider recommendation and patient screening adherence (Peterson et al., 2016). However, the study also stressed that the effectiveness of such relationships is dependent on interaction quality and the content of screening recommendations. Quality conversations addressing patient’s concerns and provider’s enthusiasm for screening improved screening adherence (Fox et al., 1994; Politi et al., 2008; Ouanhnon et al., 2023). Additionally, equipping providers with communication tools can increase screening uptake (Giveon and Kahan, 2000; Price-Haywood et al., 2014). These solutions should be considered to leverage women’s existing trust in Singapore’s HCPs.

#### 4.3.2 Social circles matter

Results from this study are consistent with previous studies that have shown the indispensable role of one’s social circle in motivating screening attendance (Dahlui et al., 2013; Documet et al., 2015; Sabgul et al., 2021; Kasper et al., 2024). In a cross-sectional study conducted in the United States, women with social support were observed to be 1.43 times more likely to be mammography screening compliant (Documet et al., 2015). In Singapore, the roles of social circle in improving screening uptake have also been explored, where the influence of one’s social circle is significant in the promotion of breast screening (Straughan and Seow, 1995; Straughan and Seow, 2000; Goh et al., 2022; Rajendram et al., 2022). As such, the engagement of diverse social groups and platforms can help to boost screening uptake.

### 4.4 Addressing concerns and promoting public confidence in RBS

As mammography screening will exist as part of RBS implementation, facilitators and limitations of mammography screening will remain relevant. Participants in this study were generally in favour of the idea of RBS, in line with findings from other studies exploring the use of RBS (Wong et al., 2017; He et al., 2018; Rainey et al., 2019; Liow et al., 2022b). While acknowledging the merits of this approach, participants expressed concerns about test accuracy and insurance implications. Resembling findings from Rainey et al., participants in this study expressed their desire to understand the scientific principles underlying the risk prediction model, results reliability, and the resulting recommendations (Rainey et al., 2019). Hence, equipping the public with information on RBS before a large-scale implementation can help alleviate uncertainties and facilitate RBS acceptance. In addition, participants were worried about potential insurance implications. Other studies exploring the implementation of RBS for various diseases have also identified insurance discrimination as a potential barrier to screening participation (Phelps et al., 2007; Koitsalu et al., 2016; Dalpe et al., 2017). Therefore, before national RBS implementation, insurance guidelines for public and private insurers must be established to safeguard the public’s interests. 4.4.1 Managing Risk Communication in RBS.

Some participants recommended that RBS be optional, as classifying individuals as “above-average” or “below-average” risk could lead to unintended consequences. A UK study found that a discordance between perceived and actual predicted risk can lead to temporary distress and rejection of information (McWilliams et al., 2023). Given that RBS predicts risk rather than providing a definitive diagnosis, careful interpretation is necessary. Participants also expressed reliance on HCPs to explain the report, emphasizing the need for personalized communication. In a Spanish study exploring HCPs’ views on RBS, HCPs called for systematic training so that they can be equipped with skills to manage patients across the spectrum, from highly anxious to disengaged, and to use RBS effectively for shared decision-making (Laza-Vasquez et al., 2022). Such training programs will be essential for the successful implementation of RBS.

#### 4.4.1 Appropriateness of recommendations

Our participants supported tailored screening recommendations and recognized the value of more frequent screening for those at above-average risk. Similar findings have been reported in Europe and the U.S., where participants acknowledged the benefits of risk-based screening adjustments (He et al., 2018; Rainey et al., 2019). Due to ethical concerns, this study did not explore women’s receptiveness to reduced screening for those at below-average risk. However, the abovementioned studies found that women became more apprehensive when such situations arose. Notably, McWilliams et al. found that while women accepted low-risk results, they emphasized that the recommendation for less frequent screening should be evidence-based (McWilliams et al., 2021). Further research is needed before implementing reduced screening for low-risk individuals.

### 4.5 Cultural sensitivity

The study lacked participants from the Malay community, which has the lowest mammography uptake compared to Chinese and Indian women (Ministry of Health Singapore, 2022). In a mixed-methods study involving Malay-Muslim women in Singapore, findings showed the role of religion in their screening decision-making (Straughan and Seow, 2000). In another study, Goh et al. discovered that among the Singaporean Malay population, factors such as perceived benefits of early detection, reminders from doctors and husbands, symptoms, a perceived divine test, and personal health responsibility facilitated mammography screening (Goh et al., 2022). Barriers included psychological effects, misinformation, religious beliefs, negative expectations, and distrust of doctors. These findings emphasize the need for culturally sensitive healthcare approaches that consider religion, age, perceived benefits, modesty, and socioeconomic factors. Tailored interventions addressing these nuances may be key to improving mammography screening rates in the Malay community.

### 4.6 Feasibility

We believe many of our recommendations are feasible with targeted implementation. Integrating RBS counseling into screening programs requires trained personnel but can be incorporated into existing healthcare services. Mobile apps and peer support networks are cost-effective tools that can enhance engagement. Primary care providers are well-positioned to facilitate RBS adoption through routine consultations, though additional training may be needed. Addressing barriers such as cost, accessibility, and health literacy will be key to ensuring widespread acceptance and effectiveness.

### 4.7 Strengths and limitations

The qualitative interview study design is a strength for an in-depth exploration of participants’ perspectives. The involvement of multiple coders and regular discussions helped ensure a high level of concordance in the analysis, enhancing the study’s rigor. Although the sample size of 11 interviewees is small and might not have captured the diverse needs of the Singaporean female population, this research provides a preliminary understanding of the common experiences and perspectives of breast cancer survivors regarding breast cancer RBS recommendation. While RBS implementation is in its infancy, the insights from this study highlighted specific areas to focus on, including the risk communication approach, understanding the reliability and benefits of RBS, and insurance coverage when designing RBS population health programs. Further exploratory work could involve conducting acceptance and feasibility research on an improved version of the RBS protocol with different stakeholders, such as healthcare professionals, women of different ethnicities and age groups, family members, and policymakers.

## 5 Conclusion

This study highlights key challenges and enablers for improving breast screening in Singapore based on the experiences of breast cancer survivors. Participants were generally receptive to RBS as a way to encourage routine screening. Effective implementation will require community initiatives to improve breast health literacy, proactive discussions by HCPs, and accessible, efficient healthcare systems to support mammography demands. Women’s acceptance of RBS will depend on further research to strengthen evidence on prediction accuracy and identify effective ways to communicate and apply risk results. By addressing these gaps, Singapore can lead in the implementation of precision prevention, setting a precedent for other countries exploring risk-adapted screening approaches.

## Data Availability

The original contributions presented in the study are included in the article/Supplementary material, further inquiries can be directed to the corresponding author.

## Supplementary material

ADDITIONAL FILE 1 Report prototype shown to participants.

ADDITIONAL FILE 2 COREQ checklist.
