# Supplementary material for: Do women with a history of breast cancer recommend risk-based breast cancer screening? An in-depth interview study
Source: Front Psychol. 2025 Apr 22;16:1414099. doi: 10.3389/fpsyg.2025.1414099 (PMC12052940; doi:10.3389/fpsyg.2025.1414099)
Supplement: ADDITIONAL FILE 1 — Report prototype shown to participants. [file Data_Sheet_1.pdf]

**Date of report**	27−Apr−2021
**Date of recruitment**	16−Apr−2021
**Age at recruitment**	40
**Date of Birth**	Apr−1980
**NRIC**	XXXXX123H
**BREATHE ID**	XXXXX

Thank you for taking part in **BREATHE** (Breast Screening Tailored for Her), a study designed to help women understand their personal risk of developing breast cancer.
On average 1 in 13 women develops breast cancer in their lifetime (Singapore Cancer Registry, 1968−2017).

Your predicted breast cancer risk is: **Above Average**

The **national breast cancer screening guideline** for your age group (40 to 49 years) is to attend **yearly** mammography screening.

These are BREATHE's additional recommendations based on your personalised risk classification:

− Get a one−stop breast care service at Ng Teng Fong General Hospital (NTFGH) or National University Hospital (NUH). This includes a consultation with a specialist to discuss how to manage your breast health and how a genetic test maybe helpful. Based on the breast specialist's recommendation, additional diagnostic screening may be scheduled. This diagnostic screening is not part of this research study. Visit any National University Polyclinic to get a referral letter for subsidized rates at NTFGH or NUH.
− Perform routine breast self−examination once every month (see next page for steps).

Personalised breast cancer risk classification − What you should know:

Although your breast cancer risk may be estimated, this prediction does not mean that you will develop breast cancer. Some women with high estimated risk may never develop breast cancer; conversely, some women with low estimated risk may still develop breast cancer. Your risk of developing breast cancer may change over time.

What else can I do to manage my risk?

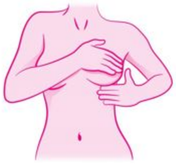

Perform your monthly breast self-examination

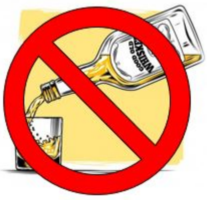

Limit or stop alcohol intake

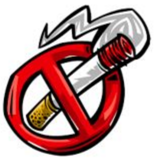

Limit or stop smoking

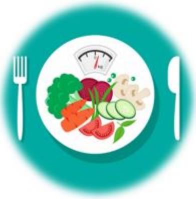

Have a balanced diet which includes whole grains, fruit, vegetables and calcium−rich food

Select healthier food choices that are lower in salt, sugar and fat

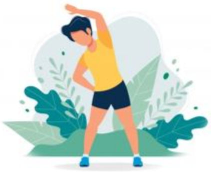

Aim to exercise for at least 150 minutes each week

For more information on genetic testing, please visit:

https://www.singhealth.com.sg/patient−care/conditions−treatments/breast−cancer−diagnosis−treatment
https://www.nccs.com.sg/about−us/our−services/Documents/NCCS%20Brochure_HBOC_A4_6%20panel%20297x210mm−Eng.pdf

If you have any questions:

Speak to our BREATHE study coordinators at +65 6716 6341,
or send an email to JHCampus_breathe@nuhs.edu.sg.

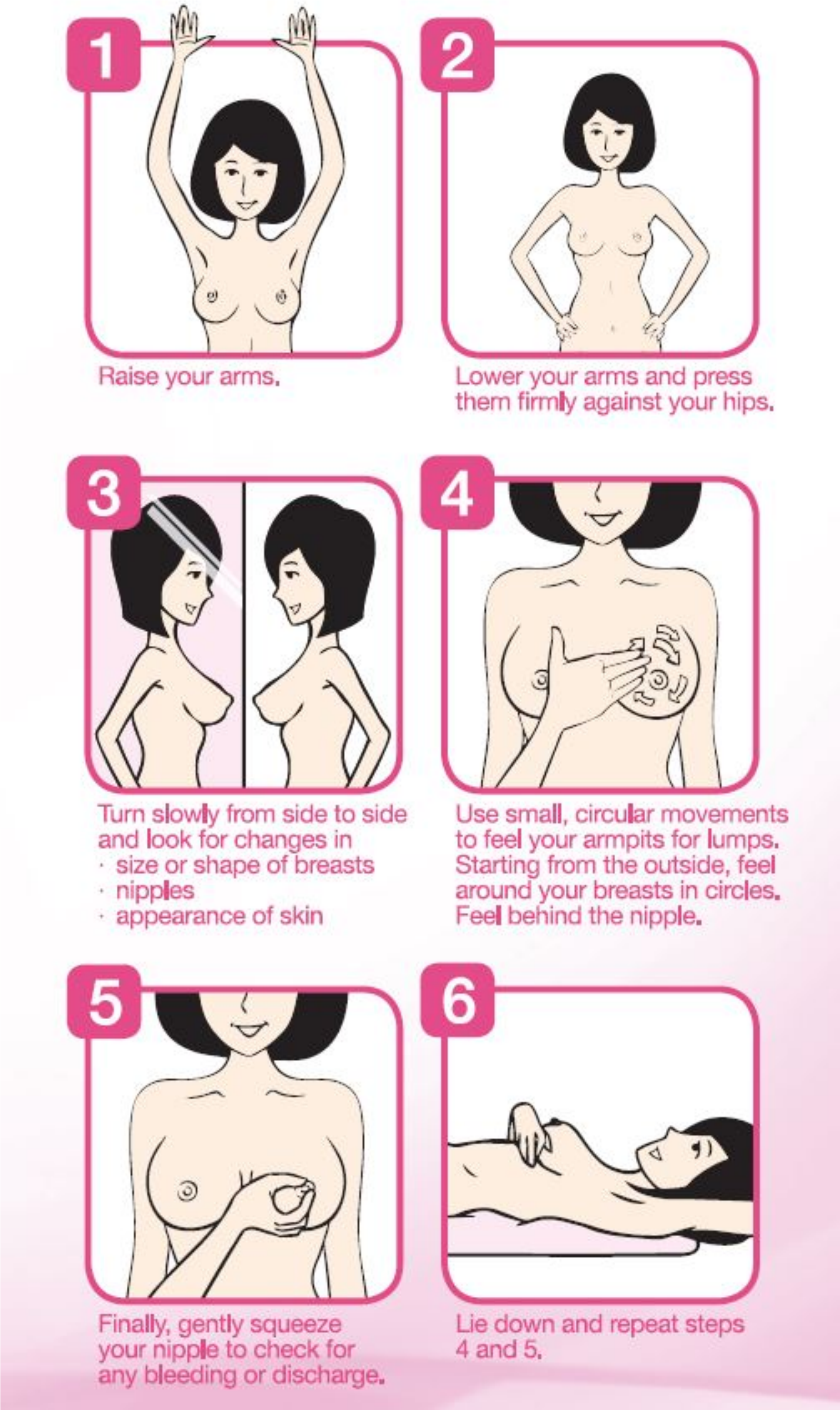

Adapted from Health Promotion Board.

Thank you for joining BREATHE.

**Date of report**	27−Apr−2021
**Date of recruitment**	16−Apr−2021
**Age at recruitment**	40
**Date of Birth**	Apr−1980
**NRIC**	XXXXX123H
**BREATHE ID**	XXXXX

Thank you for taking part in **BREATHE** (Breast Screening Tailored for Her), a study designed to help women understand their personal risk of developing breast cancer.
On average 1 in 13 women develops breast cancer in their lifetime (Singapore Cancer Registry, 1968−2017).

Your predicted breast cancer risk is: **Below Average**

The national breast cancer screening guidelines for your age group (50 years and above) is to attend mammography screening **once every two years**.

These are BREATHE's additional recommendations based on your personalised risk classification:

− Continue with mammography screening once every two years.
− Perform routine breast self−examination once every month (see next page for steps).

Personalised breast cancer risk classification − What you should know:

Although your breast cancer risk may be estimated, this prediction does not mean that you will develop breast cancer. Some women with high estimated risk may never develop breast cancer; conversely, some women with low estimated risk may still develop breast cancer. Your risk of developing breast cancer may change over time.

What else can I do to manage my risk?

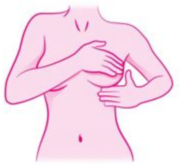

Perform your monthly breast self-examination

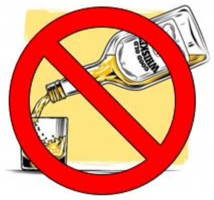

Limit or stop alcohol intake

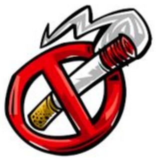

Limit or stop smoking

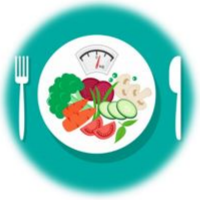

Have a balanced diet which includes whole grains, fruit, vegetables and calcium−rich food

Select healthier food choices that are lower in salt, sugar and fat

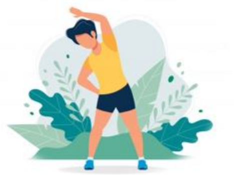

Aim to exercise for at least 150 minutes each week

If you have any questions:

Speak to our BREATHE study coordinators at +65 6716 6341,
or send an email to JHCampus_breathe@nuhs.edu.sg.

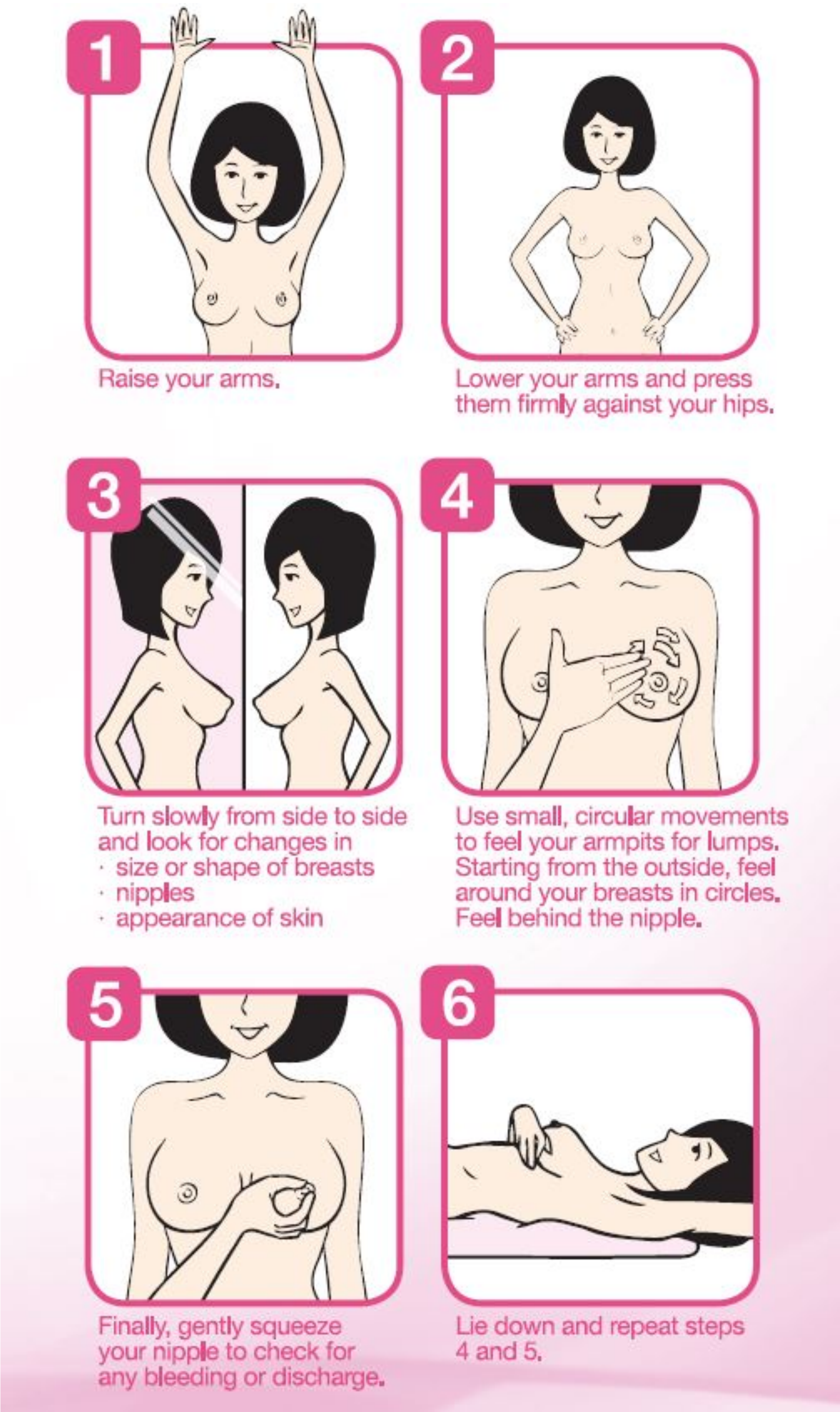

Adapted from Health Promotion Board.

Thank you for joining BREATHE.
