# Supplementary material for: Do women with a history of breast cancer recommend risk-based breast cancer screening? An in-depth interview study
Source: Front Psychol. 2025 Apr 22;16:1414099. doi: 10.3389/fpsyg.2025.1414099 (PMC12052940; doi:10.3389/fpsyg.2025.1414099)
Supplement: Supplementary file 3 [file Table_1.pdf]

Supplementary Table 1. Interview guide for in-depth interviews.

**Questions**	**Prompts (if any)**
Current screening related	
Do you know of any breast screening programs?	Can you elaborate on the program?
How old were you when you were diagnosed with cancer?<br>How was your cancer detected?	
(If cancer detected through routine mammography)<br>Prior to being diagnosed, how often did you attend screening?	
How did you find the mammography experience?	Was there enough information provided? Overall, were you satisfied or unsatisfied with the experience?
(If cancer was not detected through routine mammography)<br>Have you attended any routine mammography screening prior to being diagnosed?	
MOTIVATION<br>What are some things that motivated you to attend screening?<br><br>Can you share what influenced your decision to receive BCS? (e.g. character, personal beliefs or lifestyle that influence your decision to receive BCS)	
BARRIERS<br>What are some challenges you have faced in accessing Breast CA screening in Singapore?<br><br>What do you think has been/will be useful in helping you and other women take up Breast CA screening in Singapore?	
Looking back, what would you have done differently in your screening approach?<br><br>With a history of Breast CA, how has that influenced the way you think about the importance of Breast CA screening	
Currently, are you following any routine screening practices? Do you adhere to them? Why/ Why not?	
Currently, there are people who attend the first screening but very few go for subsequent ones.<br>What do you think are some possible barriers preventing women from attending regular screening?	How can screening programs be improved?<br><br>What further information would be useful/ should be provided prior to/ during/ after screening?<br><br>Do you think false positives can possible be a barrier to screening adherence?<br><br>Do you think the fear of finding out one’s cancer diagnosis prevents people from attending screening?
Among your friends, what are some screening trends that you have observed? From your pov, what are some factors that might have contributed to their adherence (or non-adherence)?	
What are some of the thoughts or feelings that you've experienced when you realise it is time to arrange a screening session?	

How do you think these thoughts/feelings <name them> affect the likelihood that you will arrange and attend a screening session?<br><br>what are some of the ways you could overcome this?	
Who do you talk to when it comes to health-related decisions, like going for screening?	
What can your family or closed ones do to encourage more healthful behaviours?/ How did your family support you in your recovery journey?	
Do you think routine screening programs are effective?<br>• Maybe can explain more about current guidelines→ Currently, the national guideline for breast screening is once a year for ages 40 to 49 and once every 2 years for ages 50 and above. Do you think this is reasonable?	
Risk-based screening related	
How much do you trust screening results?	
Based on your previous experiences, were the screening reports sufficient for you? If not, what kind of information should be included in screening reports so that it would be more useful for other people?<br><br>If there are calculators to see your breast/ other cancer risk level... do you think it would be more of a benefit or a hindrance? (don't mention genetics until later eg. What if this high risk is based on genetics?)	
What are some things that come to your mind when genetic testing is mentioned?	
Do you think that it is appropriate/good for screening programs to include results on genetic risk?	Why or why not?
What are some factors that should be noted if genetic risk is included as part of screening outcome?	What are some barriers that might prevent people from going for GT?
What are the advantages of knowing your personal risk of getting breast cancer and have it as part of your screening result?	What about disadvantages?
SHOW REPORT<br>This is an example of a newer version of a mammography screening report. What do you think about it?	
	And how would your attitude and behaviour change?
Should specific managements be recommended for each risk group?	
How would you feel if you got a report saying you are at "below average risk"?	
How would you feel if you got a report saying you are at "above average" risk?	
Conclusion	
(Brief Conclusions + summary)<br>Is there anything else anyone would want to add?	
