# Supplementary material for: Do women with a history of breast cancer recommend risk-based breast cancer screening? An in-depth interview study
Source: Front Psychol. 2025 Apr 22;16:1414099. doi: 10.3389/fpsyg.2025.1414099 (PMC12052940; doi:10.3389/fpsyg.2025.1414099)
Supplement: Supplementary file 4 [file Table_2.pdf]

Supplementary Table 2: Study codebook

Coding scheme of facilitators and barriers to breast cancer risk-based screening guided by the Health Belief Model

**Research Question:** What are the perceived barriers and facilitators of breast cancer risk-based screening in women with history of breast cancer?			
**Health Belief Model constructs**	**Codes**	**Codes meaning**	**Example quotes**
**Perceived susceptibility**<br>This relates to women's understanding of breast cancer, their perception of their own and others' likelihood of developing breast cancer, and their interpretation of influential factors (e.g., lifestyle, family history, age) contributing to this likelihood.<br><br>Inclusion criteria: women's understanding of how breast cancer develops.<br><br>Exclusion criteria: women's knowledge of the effects of dealing with breast cancer	Knowledge of breast cancer and/or risk factors	Women's knowledge of breast cancer risk factors and how breast cancer develops.<br><br>Inclusion criteria: Mentions of knowledge of breast cancer (e.g., pathophysiology, risk factors, BRCA genes) with/without link to self-perceived risk. This includes knowledge of breast cancer relating to other women.<br><br>Exclusion criteria: Mentions of low/high self-perceived risk without clear reasons linked to knowledge of breast cancer (e.g., self-perceived risk changed because they know of someone's diagnosis). This would be double coded under 'perceived susceptibility - Self-perceived breast cancer risk' and 'Cue to action – social support'.	*F7: In my case, I had two kids. I don't smoke. I don't drink. I breastfed both kids [for] nineteen months. I have breast cancer. I had no symptoms at all. So it's really like that shit luck type of thing right? So perhaps education should start earlier, to tell people that it happens. You cannot think that it won't happen to you.*
	Self-perceived breast cancer risk	Women's judgment of their own and/or others' chances of having breast cancer based on their knowledge of risk factors, interactions with others and personal beliefs.<br><br>Inclusion criteria: Women's interpretation of their breast cancer risks <u>before</u> considering risk-based screening results.<br><br>Exclusion criteria: Women's interpretation of their own breast cancer risks as a response to hypothetical risk-based screening results.<br><br>Double code with "Perceived susceptibility - Knowledge of breast cancer and/or risk factors"	*F1: Let's assume those women are in their 40s, right? Because that's why they're going to do it, and they probably have family commitments. They probably have work, trying to get time out is difficult. Everyone thinks it won't happen to them right? Especially if you don't have family history, you'd think "It's not a risk. It's okay. I skip it one year."*<br><br>*F11: I didn't really think that I would get cancer because I was, you know, below 40, and we don't have family history of cancer, and then I was eating healthy, and I was active. So I thought I was doing everything I could. I mean cancer was*

		for those who attributed their self-perceived risk to their existing knowledge of risk factors.	*never on my mind, to be honest, I thought I was living healthy.*<br><br>*F6: But a lot of time, uh, I think most people feel that..no lump, no pain, it's like, there isn't a need to do that yearly screening.*
**Perceived severity**<br>This relates to women's perception and experiences on the seriousness of breast cancer and the negative impact breast cancer (E.g., undesirable treatment side effects) can bring to women's and their families' lives.	Workability	Women's difficulties in retaining their jobs or returning to the workforce after getting breast cancer.	*F5: So right now, I'm currently, I'm currently not working, because chemo[therapy] was very tough for me so I can't work and do treatment at the same time.*
	Loss of identity	The struggles women face in losing their sense of femininity and identity due to breast cancer. (e.g., loss of breast and hair from cancer treatments).	*F7: I also had a few friends whose mother-in-laws would rather die because they outright just told the daughter in law 没有胸部不是女人 (no breasts, not a woman). So yeah, I've had personal friends who had weird ideas as to why they didn't do the checkup.*

	Financial impact	Women's and/experiences of the financial implications upon diagnosis of breast cancer (e.g., treatment costs).<br><br>Exclusion criteria: Mentions of the cost of breast screening or fear of medical expenses that hinder women from regular breast screening. This would be coded under 'perceived barriers' and 'financial disincentives'.	*F10: But I thought that sometimes, because people are also scared of the costs involved after they are diagnosed.*<br>*ZL: Oh like the treatment costs?*<br>*F10: So I felt that probably, you know, getting themselves well insured is also very important.....*
	Emotional distress	The emotional burden that women struggle with when dealing with breast cancer (e.g., feeling isolated/not understood).	*F5: I think when we have a problem right, it's always easy for people to say that they understand, they understand. But honestly, they will not understand unless they've encountered this situation themselves.*

	Access to healthcare	Difficulties encountered by women in receiving medical treatments due to having breast cancer (e.g., dental, fertility treatments).	*F5: The care from [breast cancer] surgery to IVF (in-vitro fertilization) is not really seamless. ....so after IVF right, they (Singapore General Hospital) gave me this hormonal injection....to protect my ovaries. My [cancer] treatment was done in NCCS (National Cancer Center Singapore).....So I need to continue on this hormonal injection because my breast cancer is hormone positive right.....And then it was a very difficult process because I need to go SGH and queue for one hour (to get the prescription), I need to bring to NCCS for the nurse to administer me.*<br><br>*F6: I went to a few dental clinic[s], and the minute they hear that I've just finished chemo[therapy], and they see my head botak (bald), straight away they say sorry they don't do. They don't even want to take me.*
	Death	Mentions of late-stage breast cancer detection, resulting in end of life.	*F10: One day all of a sudden, I found this very hard lump on my breast, which I never discovered a few days ago. So I thought I gained weight. So one week passed, second week passed, and it was still there, so that really kind of a bit not right....But you know what is it? I am (was) too busy to do anything until I bumped into one of the colleagues who shared [that] another colleagues' wife passed on from that (breast cancer).*
**Perceived benefits**<br>This relates to women's understanding, beliefs and	Early detection	This relates to women's awareness of the advantage of risk-based screening in helping them to identify their family members' risks,	*F10: I feel that it is important to know. So at least, you know that if you have a high of getting cancer, do something about it,*

perceptions on the advantages of participating in risk-based screening, including genetic testing (e.g., oncotype dx test).<br><br>Inclusion criteria: women's knowledge on practical benefits (e.g., earlier detection and better prognosis, motivating healthier lifestyle habits, supporting decision-making in financial planning and treatments) that can be reaped from participating in risk-based screening.<br><br>Exclusion criteria: interpersonal and situational influences (e.g., social support, know of someone with cancer, convenience etc.) that trigger women's inclination to participate in risk-based screening. These would be coded under 'cues to action'<br><br>Women who have explained the practical advantages of risk-based screening as a result of life experiences and interactions with others will be double coded in 'perceived benefits' and 'cues to action'.		supporting early detection and treatment and increasing survival rate.	*by, example, go for early screening to ensure that you get the early detection.*<br><br>*F8: Um early detection....you improve your chance of recovery a lot better. If you can do something about it when you detect it early, then it's a higher chance of full recovery lor. So I just make sure I screen regularly.*
	Initiate actionable steps	• Risk-based screening<br><br>This relates to women's experience of how risk-based screening has helped them and/or others in making informed, preventative decisions tailored to their profile. It also includes women being more proactive in taking charge of their health or related issues (e.g., motivated to attend screening, improving lifestyle, financial planning) after knowing their risks.	*F5: ...If, if I myself know the, know I'm at risk at certain cancers then I would definitely improve my lifestyle lah.*<br><br>*F9: Maybe it is good in a sense, because then, maybe that person has already gone through the check right? And then confirm that, yeah in that moment in time she has not have any breast cancer yet. But then her risk is above average, right? So then after she know that...then I think that will kind of like motivate her to do regular mammogram.*
		• Genetic testing<br><br>This relates to women's experiences of how genetic testing has helped them and/or others inform decisions that are unrelated to risk-based screening (e.g., treatment decisions, inform them of recurrence rate, oncotype dx test).<br><br>Exclusion criteria: Does not include any genetic tests that lead to preventative measures.	*F11: As someone who has been diagnosed. Obviously, for me, it's very important, because you know, the goal is for it not to recur again. I mean, I wouldn't want to go through it. Um, so, if, and the reason why I did the genetic testing is, I wanted to know what would be the best treatment for me, moving forward like you know. ...Then I'd want to get the best treatment possible. And hopefully the genetic testing would help, um, help my doctors and me find out the best treatment.*<br><br>*F3: But it's only because the screening is done, I could make the decision for the*

			treatment plan easier, to decide what I want to do.
**Perceived barriers**<br>This relates to women's breast screening experiences (e.g., unpleasant interactions with healthcare professionals, misdiagnosis) and/or beliefs about the associated barriers (e.g., inconvenience, expenses, uncertainty about results accuracy) involved that may impede them from participating in risk-based screening.	Poor accessibility to mammography services	Difficulty in accessing mammography services due to time (e.g., long waiting time, lack of breast screening leave), convenience (e.g., location, difficulty in booking appointments), and pandemic-related restrictions.<br><br>Inclusion criteria: Responses indicating that significant extra effort is required to attend screening due to the lack of a specified convenience.<br><br>Exclusion criteria:<br>Nonchalant attitudes or mentions of the lack of importance of screening without reference to inconvenience will be coded under 'Priorities'	*F1: If you schedule something 5 months down the road, you are not going to go because something will come up. The process needs to be easy and simple.*<br><br>*F10: The challenge, maybe sometimes the accessibility of the area. Like you know, it (the screening) could be at this place, they need to travel to certain area that they might not know where it is for example, or not convenient enough for them, and eventually, (they) just procrastinate, nevermind, don't do.*
	Priorities	Breast cancer and/or preventative measures of breast cancer (E.g. screening, healthy lifestyle?) being regarded as not important / less important than other aspects (e.g., children/work commitment/time/busy) of their lives based on personal values and experiences.<br><br>Exclusion criteria: Mentions of screening being difficult to access without any references to lack of priority or unimportance. This will be coded under "Perceived barriers - Poor accessibility to mammography services".	*F4: They don't think that they will get it, or it's just not a priority, and they are more worried about other things. And of course, life obstacles, they are so busy with work, they have children and all sorts of things.*<br><br>*F5: Hmm. I think number one definitely Singaporeans [are] very busy right, if you, a lot of commitments, work commitments, personal commitments. So I think probably they would see this (pause) as something that is… least of a priority.*
	Financial disincentives	This relates to women's concerns on the financial costs that breast screening entails (e.g., screening cost, impact of personal insurance coverage, lack of subsidies from companies/ government, and the absence of monetary incentives) which can discourage them from taking part in breast screening.<br><br>Exclusion criteria: Excludes treatment costs/ loss of income due to breast cancer. These will be	*F3: One thing is, it can be quite costly and if you're going through the subsidised route - the public sector route, it can be quite time consuming. ...That's what I see around me.*<br><br>*F5: The public (healthcare) side, I think they usually have very basic screening and if you want to have more comprehensive screening it really*

		coded under "Perceived severity- Financial impact".	*depends on your family history and all those before it is subsidised. So I think that's also part of the reason why people don't go for health screening on a yearly basis because it's pretty expensive...uh outside (of the public healthcare).*<br><br>*F10: I feel that the cost of it is also something that would deter people to go for this genetic testing unless necessary.*
	Physical features	This relates to women's physical features (E.g., breast size, breast density) that shortchange their breast screening experiences (E.g., takes longer time to screen, difficult to detect tumor, discomfort).<br><br>Exclusion criteria: excludes description of painful procedure/fear of pain without mentioning breast size.	*F2: I was very wary of mammograms….a doctor said something to me like "You have very dense breast tissue. So mammograms are going to be useless, because, like everything looks the same, they can't differentiate dense breast tissue versus something that's actually potentially dangerous." So…that kind of always stuck with me like mammograms are never going to work on me anyway.*<br><br>*F7: So my friends who are flat chest, more flat chested they do complain, they say, oh, it's a very, very painful experience for them, because literally you have the nurse elbowing them forward to get as much meat onto the plate.*<br><br>*F10: When you buy those health screening packages they include….breast cancer screening as well as… all this other screening, but just so happen…there were twice that when I went for such screening, I was menstruating. Then they will tell you that don't do it [mammogram screening] because it won't be accurate, or you are not able to do it. So it's always happened*

			and then I never had a chance to go back and do it again.
	Screening guidelines	This relates to level of awareness of national screening guideline or different screening recommendations from different health institutions and/or consideration of certain factors (e.g., age, menstruation, and breast density) that limit women’s access to breast screening.<br><br>Inclusion criteria: includes women’s decision not to attend screening when they have not reached the recommended age for screening. This also includes confusion that can arise due to differences in screening recommendations across private and public healthcare institutions.	*F1: So no, because the screening recommendations are to start for over 40, which is exactly what I did, and before that, there’s dense breast tissue. So that's why it's not recommended, so I don’t think screening earlier or more frequently would have changed that.*<br><br>*F3: (Did not attend breast screening), because breast screening, mammogram only start at the age of 40. So I was below the age. So it wasn’t something that was offered. But they do remind us, I mean, there are always guidelines to say that every month you are supposed to do your self-check to ensure that everything is normal to you.*
	Negative experience with healthcare professionals	This relates to poor experiences in healthcare services involving healthcare professionals and/or doctor’s clinical judgment that deterred women from attending breast screening.<br><br>Inclusion criteria: includes women’s decision not to go for subsequent breast screening because of negative past experiences/recounts by others. Also includes doctors’ misdiagnosis and decision-making that prevents women from accessing breast screening.<br><br>Exclusion criteria: excludes missed opportunities for breast screening due to standard operating criteria/procedures (e.g., age/menstruation) that are not decided by an individual healthcare professional.	*F6: …I was misdiagnosed actually…I ask the doctor to give me mammogram, or you know, ultrascan. But then he say no need, so I think the doctors also need to be educated much as they are given the guidelines, you know, women with lumps, and whatever actually with or without lumps 40 years old and above should go and do mammogram at least or ultrascan, but he flatly rejected me… so the screening process…is the responsibility …not just on the patient itself. It’s also doctors need to be educated, and doctors need to follow the SOP (standard of procedure)…. And especially if this women,...come and see you…already got a lump,...You should also ask the patient to go and do mammogram or ultrascan.*

	Concerns on the reliability of screening results	This relates to women's knowledge/experience on the inaccuracy of breast screening results (e.g., false positives), and general concerns around the validity of risk estimates which could hinder women from participating in risk-based screening.	*F10: You know false positive can be roller coaster ride to many girls. You know that that that anticipation while waiting for the results, because when you first found "oh, shit, like, you need to go for biopsy for example…. Oh, no", you know biopsy can also be a very traumatizing experience.*<br><br>*F9: So I mean that that information must be, you know, trustworthy…..... How accurate is it? So, I mean, if let's say it is something that is, you know, the accuracy is maybe like 50-50, then I will have my reservation about doing such a check.*
	Discomfort	When women mention that screening is uncomfortable/ painful based on their knowledge or own experience(s) without reflecting their capability to withstand it.<br><br>Exclusion criteria: Excludes women expression of how tolerable the pain (experienced or perceived) from mammography is to them. These will be coded under "Self efficacy- Pain".	*F3: Before you go for mammogram you hear a lot of people telling you that it is very painful, it is very tough, it's just not comfortable.*<br><br>*F4: If someone dread(s) doing something right, the odds of them doing it regularly is lower, right? To them, it's like so painful, so stressful don't want to do it.*
	Lack of social support	This relates to the lack of conversation among women and their social circle regarding health screening.	*F6: It all depends. Because, if let's say, your parents are the highly educated ones, they would have already inculcated the educated people kind of thinking whereby you will be very… you will be taught to be self responsible for your own health. But if you come from a family background where your parents are… not the educated type, their thinking would be: "Nothing wrong why you go to hospital? Nothing wrong why you go take blood? Nothing wrong why you go screening?" You know, so that is the kind of upbringing*

			you have right, then, over the years you will not have that kind of thinking of wanting to go and do health screening. So I think it's the environment and also the people you mix with.
	Negligence from low-risk results	This relates to the false sense of security reactions women could have when they think about hypothetical below average breast cancer risk result, which may reduce their motivation to attend screening and/or maintaining healthy lifestyle.<br><br>Exclusion criteria: Excludes low compliance to regular mammography screening due to having cleared from it in the past. These will be coded under 'Perceived susceptibility - Self-perceived breast cancer risk'	*F6: So actually, by putting "below-average" right, [it] is actually deterring people from doing (going for regular screening), because people will very conveniently think that "Oh, that means my chances of getting it (cancer) is very low. Then no need [to screen]. Wait until the lump comes out then I go [for screening]." Definitely, a lot of them will have this kind of thinking.*
**Cues to Action**<br>Life events, social interactions (E.g., breast cancer support group, spousal support), or physical symptoms (e.g., pain, lump) that enable/trigger women's readiness to participate in risk-based screening/genetic testing.	Social support<br><br>This relates to the role of various social mediums in providing emotional and practical support (E.g., educating women about breast cancer, appointment booking) that facilitates women to attend breast cancer screening<br><br>Inclusion criteria: Includes sharing from women or by other social mediums women are in about the importance of or information on breast	• Family influence<br><br>Women's experiences in receiving/giving support from/to family (e.g., genetic testing for family's health and financial planning, assistance in booking mammography services, emotional support) that promote/can promote participation in risk-based screening.	*F7: My mother has a pretty big cup [size], and then she has a regular cyst developing in her breast, every now and then. And they're fairly big. So in my family we have talked about mammogram quite often, so I already have an idea about it, because my mom goes for it.*<br><br>*F4: But that said, i think people with daughters, might want to (go for RBS), because if they have daughters they would want to know whether it's genetic. But I have no children, so it could be a perspective thing.*
		• Friend Influence<br><br>Women's experiences in receiving/giving support from/to friends (e.g., practical help/persuasion to sign up for mammography services, physical	*F4: Actually, we know about mammogram, at 34 years old we did. We have a friend who [when] in her 20s she went for a check-up and the doctor actually sent her for a biopsy because the doctor felt a lump. So in our 20s we were already*

	cancer screening and prevention.<br><br>Exclusion criteria: Excludes social support on cancer treatment/recovery journey that are not related to risk-based screening. Feeling of being socially isolated as a result of having breast cancer would be coded under "Perceived Severity - Emotional distress".	accompaniment, emotional support) that promote/can promote participation in risk-based screening.	*exposed to this knowledge, we know about mammogram and breast cancer.*
		• Workplace/colleagues<br>Women's experiences and beliefs about the role companies/colleagues have played/can play in promoting participation in risk-based screening.<br><br>Inclusion criteria:<br>Includes interpersonal relationship and workplace initiatives (E.g., health screening subsidies, health talks in workplace) that encourages women's participation in risk-based screening.	*F2: I just went along knowing I had a lump for at least six months, as far as I can remember. I only actually went to a doctor when one of my colleagues at the office was diagnosed with breast cancer, and she was younger than me. That's what shocked me into the system. Like to go and get it checked out.*<br><br>*F10: I just did one recently… So it is partly because my company gave us $350 yearly to go for such a screening, so that actually helps in a way to offset the cost of health screening, …. actually encourage me to go for the checks.*
		• Breast cancer support groups/ other women<br><br>Women's experiences in receiving or giving encouragement or advice to or from other women with lived experiences and/or common concerns about breast cancer, that promote participation in risk-based screening.	*F11: …So when I started joining BCF (Breast Cancer Foundation), for example. Obviously, they'(the members) are more aware with breast screening since they went through breast cancer. So I get more information from these kinds of groups as compared to you know, when i'm just with, when i'm talking to my friends who haven't had it. So yeah, more substantial information from social communities who have gone through it.*
		• Social media<br><br>The use of social media as a tool for health messaging to increase awareness and promote participation in risk-based screening.<br><br>Double code with "Social opportunities" (depending on the stakeholder mentioned) if	*F10: I shared on my social media, my cancer journey, so that actually kind of inspired or motivated most of my girlfriends to actually go for the screening... I actually shared my journey, like how mammography works, and it's actually not that bad. ….So I think that actually helps people debunk this myth*

		participant mentions (stakeholder) increased awareness through social media.	*that it's painful, and to go for it [mammography].*
	Symptoms	The presence of physical symptoms and/or discomfort (E.g., lump/pain) which triggers participation in risk-based screening.	*F11: I detected it myself, I mean I found the lump, and then the next day I went straight to the GP. And then the GP sent me straight to the breast surgeon, and then, after a few days, that's when I got the diagnosis.*
	Insurance coverage	The impact of having insurance (E.g. reduced cost/free health screening packages and lower financial distress in the event of diagnosis) which motivates women to attend screening.	*F7: And then I think for a lot of them is insurance. They're not sure, because in my case, after I was diagnosed, I think just the medication itself has easily hit 250K. Operation, medication. So from the day I was diagnosed til now it's already 250K. And it's all covered by my insurance. But if you're talking about lower income people where they're going to cough up that you know through insurance that their basic insurance will not be enough.*<br><br>*F10: But I thought that sometimes, because people are also scared of the costs involved after they are diagnosed…So I felt that probably, you know, getting themselves well insured is also very important.*
	Healthcare infrastructure<br><br>This relates to how the healthcare infrastructure (healthcare professionals, government) facilitates women to attend breast cancer screening.<br><br>Inclusion criteria: Includes suggestions on	• Healthcare<br><br>This relates to recommendations made from healthcare to participate in breast screening (e.g. Post-partum check),<br>positive experiences with healthcare professionals (e.g., family doctor, radiologist, psychologist) and/or the healthcare system that builds trust and promotes inclination to participate in risk-based screening.	*F9: I mean for myself, right where the first time that I did the mammogram is actually through the recommendation of the doctor who actually did my usual medical checkup.*<br><br>*F1: And also you might go to a GP (general practitioner) for a cold. But then the GP should just ask like "When did you last attend a mammogram?"... So you know, maybe their GP can check in on these for them.*

	how the healthcare system can be improved (e.g., national efforts to increase education).<br><br>Exclusion criteria: Excludes mediums where breast cancer screening is encouraged through non-healthcare related social/interpersonal relationships and support.	Exclusion criteria: Interactions/experience with healthcare professionals that hinder women's involvement in risk-based screening. This would be coded under 'Perceived Barriers – Negative experience with healthcare professionals.	
		• Public education<br><br>Women's experiences and beliefs on how the different stakeholders (e.g., Ministry of Health, nonprofit organizations) play/can play a role in increasing their breast cancer knowledge and urge participation in breast screening.<br><br>Inclusion criteria: Includes any mentions of educational/ public awareness campaigns without specifying a stakeholder.<br><br>Exclusion criteria: Any comments mentioning that doctors/ physicians should recommend screening/ urge the public to go for screening. Those would be coded under 'Cues to Action-Healthcare Infrastructure-Healthcare."	*F5: I think it (screening) really depends on the person's character also…I think education is very important and of course. I think we need to highlight [to the public] that you know breast cancer is very highly treatable, it is not something that you know... when you have it… uh you would immediately die.*<br><br>*F10: So I felt that outreach program should always encourage them to let them know that sometimes [after] early diagnosis, you can live a very fufilling [life], like life is normal. It doesn't mean that once you have the big C, your lifestyle will change or you will die you, you will have to go through chemo, it is not true.*
		• Accessibility to mammography services<br><br>The ease of accessing mammography services that promote participation in risk-based screening.<br><br>Inclusion criteria: Includes existing/suggested external measures/systems in place such as convenient locations, fast/prompt waiting time, hassle-free process, cues/reminder to book mammogram appointment, ease of appointment bookings, subsidies.	*F3: Some estates are already working with singhealth to get their Mammobus into the estate so that their residents could get it easier and at a cheaper rate.*<br><br>*F10: I thought that maybe the clinic they went for...or the polyclinic, sometimes they will send reminders like example [for] your vaccine.....I thought that would be a good way of encouraging more (attendance) because sometimes you just lose track of time.*

		Exclusion criteria: Excludes mentions of attending breast screening without specifying reasons that facilitated them to do so. Motivation to attend breast screening due to knowledge of breast cancer will be coded under "Perceived susceptibility – knowledge of breast cancer and/or risk factors". Non-external measure such as support/assistance rendered by others (e.g. helping to book appointments) will be coded under "Cues to action – social opportunities"	
**Self-Efficacy**<br>Women's level of confidence or personal beliefs in their ability to adhere to screening guidelines, interpret and accept risk results, and cope with diagnosis.	Pain	Experiences of women's ability/inability to overcome painful procedures involved in risk-based screening.	*F6: And also they (nurses) are very courteous. They are very nice. They try to make you less uncomfortable as possible, but you like it or not, a scan is a scan, you know. Whatever has to be done has to be done. You know, so of course it will be a little bit painful when they press that kind of thing. But, I mean. This part and parcel of the deal lah, you have. You want to do test you have to bear with it lor, ya.*<br><br>*F1: It is definitely painful. I think the technician conducting that was trying to be as sensitive as possible. But of course, it takes several tries sometimes to get accurate images. So it's time consuming, and it's uncomfortable, and need to stand in a weird posture and of course it's pain.*
	Fatalistic attitudes	Women's pessimistic attitudes towards the possibility of changing their health situation with the perception that whatever is meant to happen will happen and that one has no control over changing their fate of dying from cancer, which hinders breast screening attendance.	*F10: Some people say that you know "what for go for all this screening?" ...I [would] rather die quickly, than you know, go through all this taking the pills and eventually dialysis and all these."*
	Fear of undesirable results	This relates to the phobia of getting cancer diagnosis/ "high risk" results and what these entails, that can be too overwhelming	*F5: I think the fear of knowing that it's (the screening result might be) cancer, the fear that we need to go through chemo[therapy]....is a very terrifying thing*

		for some women to process that some would rather not attend risk-based screening.<br><br>Inclusion criteria: Includes emotional reactions (E.g. anxiety/fear/scared) that hindered women from attending risk-based screening without specific reason.<br><br>Exclusion criteria: Excludes women's concerns on impact of getting risk results/diagnosis (E.g. cost of treatment/ treatment side effects/ employability etc.), these would be coded under "Perceived severity"	*lah. So...I think that's the reason why people refuse to accept the fact that... "I got a lump I need to immediately go (for checks)."....They just don't want to know bad things, and then they delay.*<br><br>*F3: As a patient, I would say if I know it (breask cancer risk) earlier, I may pay more attention to it. But as someone who have never been diagnosed, or never really know much of it beforehand, knowing this risk factor may put on additional fear and events into your life.*
	Ability to interpret health screening reports	Women's experiences of interpreting health/risk report results (E.g., difficulty understanding jargons used in reports) and areas of need (E.g., having a healthcare professional to explain, information on actionable steps) in facilitating their understanding of health/risk report.<br><br>Exclusion criteria: Any suggestions on the layout of risk report prototype, without specifying how they can improve understanding of the report.<br><br>Double code with "Others- Feedback on prototype risk report" if participant mentions that certain features of the report (E.g., medical jargons, suggested lifestyle, etc) are difficult to understand.	*F6: For reports right, to be very honest, I think most of us, who are not medically trained right, whatever reports you give us, we read already also don't understand. So that report to us ah, it's just for claiming insurance purposes....Because all the terms inside so chim (difficult). Who knows?*<br><br>*F11: I mean [as for] the information per se on the paper (report), I would say it's substantial. But I think what makes it enough is after I get to speak with a doctor, and when everything is explained in layman terms what exactly the paper means.*<br><br>*F4: When you see a medical report, wah, how do I read that? I find that medical reports are very difficult to understand, because we are not medically trained. Sometimes when you go and see the doctor they will just put the report there and use the pen and circle here and circle there. I don't understand, I just nod along. So good doctors will explain to you. Those*

			busy doctors won't explain in depth… So these medical reports doctors see is second nature [to them] because they are so used to it.<br><br>*F10: The report you know, nowadays, Google is everything you need. Sometimes, before you even enter a doctor's clinic, you just go and Google.*<br><br>*F8: Actually, the mammogram report (referring to the prototype) is more for like, the specialist to explain to us. Because most of the reports is using jargon, so a layperson will not understand fully what it's about. So we still need the doctor, the specialists, to explain to us thoroughly…..however detailed or not detailed the report is, to us, it's like a different language.*
	Health-conscious type	This relates to women inclination to participate in screening because they are proactively concerned about their health and wellbeing influenced by their personalities, environment, and/or personal values.	*F5: I don't know what triggered me (to self-exam) or maybe I don't know… I'm paranoid in nature.*<br><br>*F7: They (her kids) are still very, very young, and I want to make sure that, you know, we can be around for a long time to see them grow up. And so that's probably the reason why my husband and I are very, very diligent in getting our annual checkup just to make sure that we have more years with them.*
**OTHERS**	Acceptability towards risk-based screening	Women's personal response to the appropriateness of implementing breast/other cancer risk/genetic risk screening at national level.	*F11: I think it is good to know what your risk profile is. But again....I'm not sure if women in general would be willing to go through this type of test. And my reason for that is, first, I don't think knowing their breast cancer risk....I don't feel that they would think that knowing their breast*

			cancer risk would be relevant to them, especially if they don't have a history. And second, I don't think they would be willing to go through the mental challenge of let's say, what if it comes back as "high risk"? How would that impact [on] their quality of life?<br><br>*F3: It (if RBS is a benefit or hindrance) actually goes both ways, depending [on] how you want to see it. As a patient, I would say if I know earlier, I may pay more attention to it. But as someone who have never been diagnosed, or never really know much of it beforehand, knowing this risk factor may put an additional fear into your life.*<br><br>*F6: It (having cancer risk calculator) is a benefit, so that we can get treatment early, and also when it (the disease) is in the early stage right, I believe our options for treatment is more. There will be more options and also there will be a good prognosis.*
	RBS implementation needs	Feedback or concerns raised by women when asked about the feasibility of nationwide RBS implementation.	*F5: Hmm...I think if you, we want to do this genetic testing you need to....the organisation need to be able to answer questions from the public...... So must be able to deal with all this lah. So what if I'm high risk? How are you going to help me, what [are] you going to do for me?*<br><br>*F10: According to my understanding, the genetic testing can vary from different medical providers. Example, the government restructured hospital may cost above SGD800 to SGD1000, but for private doctors right, they may charge up*

			to SGD2000. So I think it is a huge cost to some people....I feel that the cost of it is also something that would deter people to go for this genetic testing unless necessary.
	Feedback on prototype risk report	Suggestions or comments given by women about the design, layout, and content, when presented with prototype risk report during interview.	*F4: [It will be good to have] some statistics like some explanation of how you determine the above-average, like why is it average, why is it above-average. It doesn't have to be here. It could be a link, or somewhere I can go for information.*<br><br>*F8: Like when it's offered to the patient, the patient must know that just because this part comes back to them as like low risk or above average risk, is just a guide lah so It's not like...if you are low risk don't think that it's like "wah clear already" for you.*<br><br>*F2: I think it (the prototype) is a very good start, and I think if any program that's put in place can empower women to look after themselves in the first place and... almost facilitating, like how to get resources if you need to. I guess that's the one thing I would add to that report. If it [the report] recommends you go for some type of screening, give them the information right there on how they [can] access it.*
	Knowledge of risk-based screening/ genetic testing	Women's understanding and/or personal experience of risk-based screening/ genetic testing.<br><br>Inclusion criteria: Includes when women mention the types of genetic testing they have used during diagnosis/ treatment when asked about their perception of genetic testing.	*F1: (What are some things that come to your mind when genetic testing is mentioned?) Whether someone has the BRCA gene or not.*<br><br>*F3: Actually, in genetic testing, when they do genetic testing, they will always tell you there's three types of result. It's either a*

			positive, negative, or unknown, where they cannot determine whether it is genetically causing or not.<br><br>*F4: So if you ask me genetic testing right, [I] will think of Angelina Jolie, because it's the example. She went for the genetic testing, and because of the results right, she remobed her breasts, and she removed her ovaries.*<br><br>*F6: I was asked to do an oncotype test to see whether...like whether chemotherapy will benefit me or not, because mine is stage one.*<br><br>*F8: From what I've read and heard is, if you are positive for that gene (referring to BRCA gene), it puts you at super high risk of developing the cancer further down. It's high enough to, for a person to just consider seriously undergoing mastectomy.*
